# Supplementary material for: Characterization of High-Risk HPV/EBV Co-Presence in Pre-Malignant Cervical Lesions and Squamous Cell Carcinomas
Source: Microorganisms. 2022 Apr 24;10(5):888. doi: 10.3390/microorganisms10050888 (PMC9144326; doi:10.3390/microorganisms10050888)
Supplement: Supplementary file 1 [file microorganisms-10-00888-s001.zip › Figures S1.pdf]

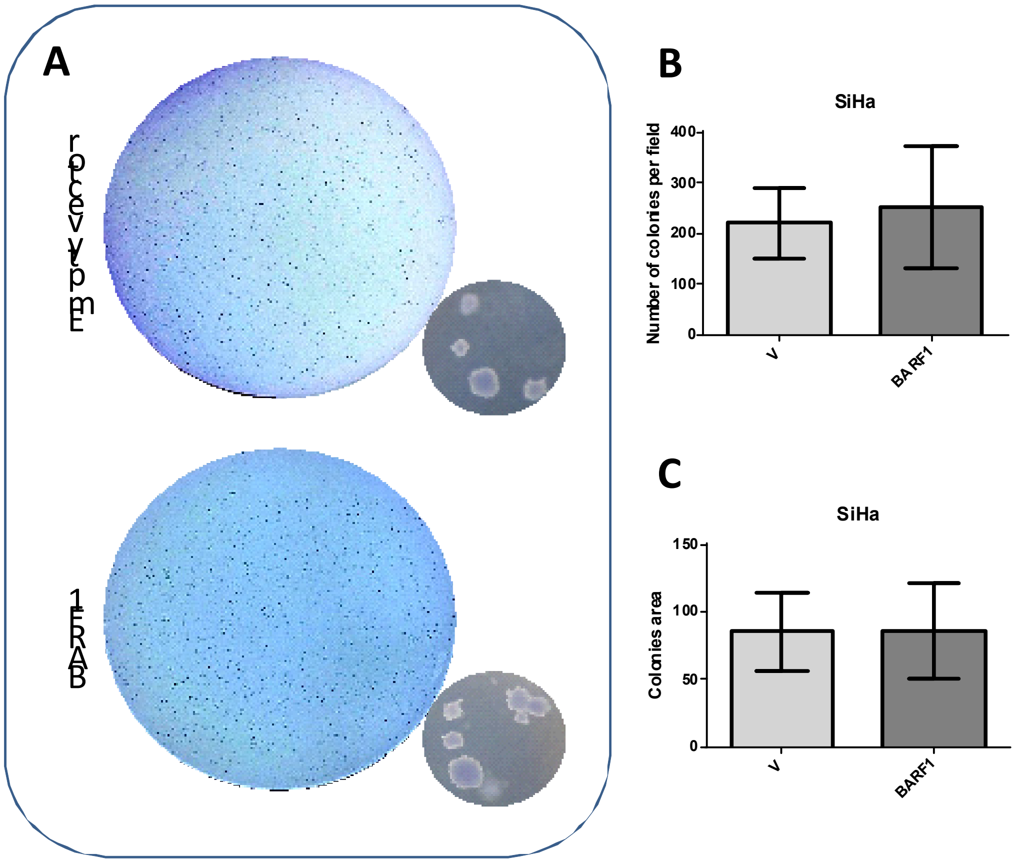

Figure S1. Anchorage-independent growth in BARF1-transfected SiHa cells. (**A**) Representative images of cell colonies formation. No significant differences were evidenced between SiHa cells transfected with empty vector or BARF1 when the number of colonies per field (**B**) or colonies area (**C**). Results correspond to three independent experiments.
