# Supplementary material for: Characterization of High-Risk HPV/EBV Co-Presence in Pre-Malignant Cervical Lesions and Squamous Cell Carcinomas
Source: Microorganisms. 2022 Apr 24;10(5):888. doi: 10.3390/microorganisms10050888 (PMC9144326; doi:10.3390/microorganisms10050888)
Supplement: Supplementary file 1 [file microorganisms-10-00888-s001.zip › Figures S2.pdf]

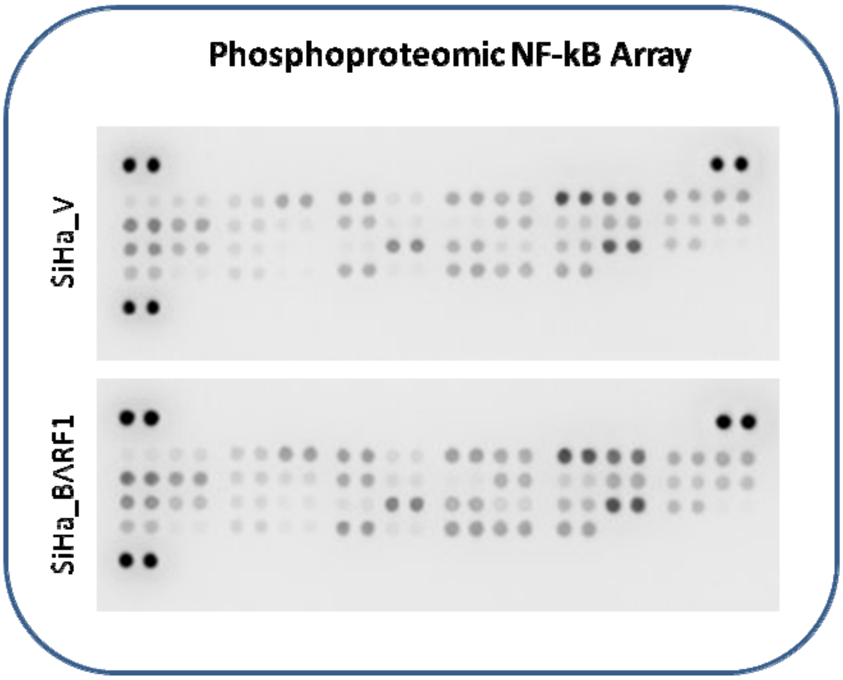

Figure S2. Protein array for NF-κB signaling pathway in BARF1-transfected SiHa cells (membranes). Conditions: SiHa cells transfected with empty vector (upper) or BARF1 (lower).
