# Supplementary material for: Characterization of High-Risk HPV/EBV Co-Presence in Pre-Malignant Cervical Lesions and Squamous Cell Carcinomas
Source: Microorganisms. 2022 Apr 24;10(5):888. doi: 10.3390/microorganisms10050888 (PMC9144326; doi:10.3390/microorganisms10050888)
Supplement: Supplementary file 1 [file microorganisms-10-00888-s001.zip › Table S1.pdf]

Table S1. Primer sequences and PCR conditions used in this study

Name	Primers (5′-3′)	Amplification conditions	Amplicon (pb)
β-globin (PCO3/PCO4)	Forward: ACACAACTGTGTTCACTAGC<br>Reverse: CAACTTCATCCACGTTCACC	Initial denaturation at 95°C for 5 min followed by 40 cycles of denaturation at 95 °C for 30 s, annealing at 52°C for 30 s and primer extension at 72°C for 30 s, with a final extension at 72°C for 5 min.	115
HPV (GP5+/GP6+)	Forward: TTTGTTACTGTGGTAGATATCAC<br>Reverse: GAAAAATAAACTGTAAATCATATTC	Initial denaturation at 95°C for 5 min, followed by 45 cycles consisting of denaturation at 95 °C for 1 min, annealing at 50°C for 2 min and extension at 72°C for 1.5 min, with a final extension for 5 min at 72°C.	155
BNRF1 (EBV)	Forward: CCTGGTCATCCTTTGCCA<br>Reverse: TGCTTCGTTATAGCCGTAGT	Initial denaturation at 95°C for 2 min followed by 40 cycles of denaturation at 95°C for 15 s, annealing at 60°C for 15 s and primer extension at 72°C for 30 s, with a final extension at 72°C for 2 min.	95
BARF1 (EBV)	Forward: CTTTCTTGGGTGAGCGAGTC<br>Reverse: CCAATAAGCACCTGCTCCTC	Initial denaturation at 94°C for 5 min followed by 33 cycles of denaturation at 95°C for 45 s, annealing at 56°C for 40 s and extension at 72°C for 45 s, with a final extension for 5 min at 72°C.	115
E6 small (HPV)	Forward: CTGCAAGCAACAGTTACTGCGA<br>Reverse: TCACACACTGCATATGGATTCCC		
β-actin	Forward: CCACACAGGGGAGGTGATAG<br>Reverse: GGGCACGAAGGCTCATT		
